# When Host’s Defenses Take a NAP: Nucleoid-Associated Proteins as Integrators of Infection and Virulence

**DOI:** 10.3390/microorganisms14102184

**Published:** 2026-09-29

**Authors:** Hali Sanogo, Marion Hoareau, Frédéric Crémazy

**Affiliations:** Université Paris-Saclay, Université de Versailles Saint-Quentin-en-Yvelines, Inserm, Infection et Inflammation, 78180 Montigny-Le-Bretonneux, France; hali.sanogo@uvsq.fr (H.S.); marion.hoareau@uvsq.fr (M.H.)

**Keywords:** nucleoid-associated protein, environmental adaptation, chromatin organization, xenogeneic silencing, stress adaptation, virulence

## Abstract

Pathogenic bacteria encounter many stresses, from their environmental niches to colonization of their host. As unicellular organisms, a rapid and efficient response is crucial for their survival. Importantly, pathogenicity depends not only on the presence of virulence genes but also on how the bacterial chromosome is organized and regulated. Nucleoid-Associated Proteins (NAPs) are small, conserved proteins described as chromosome architects as their DNA-binding abilities allow them to remodel the nucleoid. Their direct impact on DNA transactions and their role as environmental sensors make NAPs key factors in adaptation, stress responses and virulence. During infection, they modulate foreign genetic material expression, transcription programs and interactions with host cells to resist harsh environments, establish colonization and counter host defenses. Here, we review the roles of NAPs following the infection process across various species, highlighting their essential and conserved functions at every step.

## 1. Introduction

Bacterial infections represent a persistent and escalating threat to human health, driven not only by the outstanding adaptability of pathogenic bacteria but also by the global increase in antibiotic resistance [1]. Pathogenic bacteria are responsible for a broad spectrum of infections in humans, a diversity that stems from their ability to adopt two fundamentally distinct lifestyles. Some remain extracellular, colonizing tissues or forming biofilms, whereas others invade host cells and persist as facultative or strict intracellular pathogens. Throughout infection, these lifestyles expose bacteria to distinct host environments, ranging from mucosal surfaces and other extracellular niches to intracellular compartments shaped by acidic pH, oxidative stress and nutrient limitation. Their ability to thrive in such diverse and sometimes hostile environments relies on tightly regulated genetic programs that coordinate responses to environmental cues [2,3]. This regulation is particularly critical for virulence, whose expression is costly and therefore tightly restricted to conditions in which it provides a fitness advantage [4]. Environmental signals are ultimately integrated at the level of gene expression, through transcriptional networks, regulatory RNAs and DNA-binding proteins.

The bacterial nucleoid is a highly organized and dynamic structure composed of DNA, RNA, and thousands of proteins, adopting a hierarchical architecture that ensures sufficient compaction to fit within the cell while maintaining accessibility for essential DNA transactions [5]. This multilevel organization results from a combination of molecular crowding, depletion forces, DNA supercoiling, and nucleoid-associated proteins (NAPs), enabling bacteria to compact their chromosome while maintaining the ability to rapidly reorganize it in response to changing conditions [6]. NAPs are key architectural and regulatory components of this organization. By bending, wrapping, bridging, or coating DNA, they contribute to chromosome compaction and influence the accessibility of regulatory regions, thereby linking nucleoid architecture to gene expression. Beyond their architectural and transcriptional roles, NAPs also contribute to DNA replication, recombination and repair, highlighting their broader importance for bacterial physiology [7,8].

The role of NAPs in chromosome organization and gene regulation has been extensively reviewed elsewhere [7,8,9,10]. We therefore briefly summarize the main functions of the major NAPs before focusing on their roles in bacterial adaptation and virulence. NAPs are commonly classified according to their predominant modes of interaction with DNA, including bending, bridging, wrapping, and coating, although these activities often overlap (Table 1). DNA-bending proteins such as HU (histone-like protein from *E. coli* strain U93), IHF (Integration Host Factor), and Fis (Factor for Inversion Stimulation) contribute to chromosome compaction by introducing local changes in DNA topology. HU is highly conserved and can bind DNA with relatively low sequence specificity, while showing higher affinity for supercoiled or structurally distorted DNA [11,12]. HU induces sharp DNA bends that facilitate the formation of DNA loops and contribute to chromosome compaction [11]. Its architectural role is supported by nucleoid decondensation observed in HU-deficient cells [13,14]. Fis and IHF show similar effects, but differ in the extent of the distortion they impose: IHF recognizes a degenerate consensus and introduces one of the sharpest bends described for a DNA-binding protein, exceeding 160°, whereas Fis binds a broader range of sites and produces more moderate deformations [15,16,17,18]. Fis abundance also varies strongly with growth conditions, linking its architectural activity to changes in cellular physiology [19].

DNA-bridging proteins provide another important mode of chromosome organization. H-NS, together with other H-NS-like proteins such as StpA, Lsr2, and Rok, preferentially associates with AT-rich and intrinsically curved DNA, features frequently found in horizontally acquired genomic regions [7,20]. H-NS binds DNA as dimers and, at higher concentrations, oligomerizes and propagates along the DNA to form extended nucleoprotein filaments [21,22]. Depending on the local conditions, these filaments can either coat DNA or interact with a second DNA segment, generating bridges between distant regions of the chromosome [21,23]. This bridging activity contributes to chromosome organization, while H-NS binding can also restrict RNA polymerase access and silence horizontally acquired genes, including virulence-associated loci [20,24]. Importantly, the balance between DNA coating and bridging depends on the physicochemical conditions of the cell, providing H-NS with a means of responding to changes in its environment [25].

Other NAPs contribute mainly through extensive DNA binding and compaction, particularly under stress. For example, Dps accumulates during stationary phase and coats the chromosome, promoting its condensation and helping protect DNA from oxidative damage [26].

NAPs contribute to bacterial adaptation by integrating environmental signals with chromosome organization and gene expression. Unlike two-component systems, which are dedicated to sensing specific chemical or physical cues through defined receptor and phosphorelay modules, NAPs do not function as dedicated sensors. Here, we use “environmental sensing” more broadly to describe how environmental cues can influence NAP function, either through their effects on NAP-DNA binding or oligomerization, or through upstream regulatory pathways that modulate NAP abundance or activity. Under stress, these changes can alter DNA topology, protect the chromosome, and modulate the expression of genes involved in survival [6]. In pathogenic bacteria, NAPs regulate virulence genes through multiple mechanisms, including basal silencing of horizontally acquired loci, coordination with secretion systems, and modulation of DNA supercoiling in response to lifestyle-specific pressures [27,28]. Their regulatory effects can further fine-tune gene expression during transitions between distinct physiological states, such as motility and biofilm formation or persistence within host cells [6,29,30].

In this review, we examine how NAPs participate in every stage of the infectious cycle, following the chronological sequence through which bacteria encounter, adapt to, and even exploit host environments (Figure 1). By tracing infection from initial contact to establishment inside the host, we highlight how NAPs act as architectural and regulatory hubs that continuously reshape the bacterial chromosome to meet the requirements needed at each phase.

## 2. Conserved Adaptive Strategies Across Environmental Reservoirs and Host Niches

### 2.1. Xenogeneic Silencing: NAPs as Gatekeepers of the Accessory Genome

Pathogens frequently acquire virulence-associated genes through horizontal gene transfer, contributing to the rapid evolution of pathogenic traits [31,32,33,34]. Such exchanges can occur in both ecological and host-associated environments where bacteria come into close contact, exposing pathogens to mobile genetic elements (MGEs) encoding potentially advantageous traits such as virulence or resistance. Newly acquired sequences must nevertheless be integrated into pre-existing regulatory networks, and their immediate expression can impose a fitness cost [35,36,37]. Xenogeneic silencing limits this cost by keeping newly acquired DNA transcriptionally repressed from the moment of acquisition. In pathogens, this provides basal control of horizontally acquired virulence genes and prevents their inappropriate expression.

Among all the NAPs, H-NS in enterobacteria and its functional counterparts Lsr2 in actinomycetes and MvaT in *Pseudomonas* spp. have emerged as central regulators of xenogeneic silencing, and are considered “genome sentinels” [28,38,39,40]. Although unrelated in sequence and structure, these proteins have evolved remarkably similar mechanisms to recognize horizontally acquired DNA. Rather than binding specific nucleotide sequences, H-NS and Lsr2 preferentially target AT-rich DNA by sensing the narrow minor groove characteristic of horizontally acquired regions through their AT-hook motif [20,41]. H-NS still displays higher-affinity sites defined by a degenerate consensus, which are themselves enriched within pathogenicity islands [42]. Despite their distinct evolutionary origins, H-NS and Lsr2 are functionally interchangeable, illustrating a striking example of convergent evolution [40]. MvaT uses a different strategy, with an “AT-pincer” motif and multiple lysine residues that interact with the minor groove and the phosphate backbone [43]. ChIP-based approaches revealed their strong enrichment at low-GC horizontally acquired DNA, with peaks often spanning several megabases, consistent with their oligomerization on DNA, a hallmark of H-NS-like proteins [28,39,40,44]. Repression would then depend on the formation of extended nucleoprotein filaments and, in some contexts, DNA-DNA bridging, resulting in inhibition of transcription [8,45,46,47,48]. The two best-characterized modulators of H-NS and StpA in xenogeneic silencing are probably Hha and its close paralog YdgT, which act to reinforce repression on AT-rich sequences encoding antimicrobial and virulence genes, notably the hemolysin operon of *V. cholerae* and the SPI-1 island in *Salmonella* [49,50,51,52,53,54,55,56]. Beyond silencing foreign DNA after its acquisition, recent work shows that H-NS can also facilitate the establishment of AT-rich MGEs by acting as a recruitment platform for transposases, thereby promoting transposon capture and insertion into the chromosome [57].

Because cells maintain a relatively constant H-NS-to-DNA ratio, the acquisition of a large AT-rich MGE can sequester host xenogeneic silencers and perturb global gene regulation [36,37]. To minimize this burden, many MGE encode their own H-NS-like proteins, which both supplement the host silencing capacity and restrict repression to the newly acquired DNA. In uropathogenic *Escherichia coli* (UPEC) and *S. Typhimurium*, StpA contributes to repression of horizontally acquired virulence genes, including fimbriae and hemolysin genes in UPEC and the SPI-1 and SPI-2 pathogenic islands [58,59,60,61,62], while plasmid-encoded proteins such as R27 H-NS and Sfh in *S. flexneri* provide additional examples of MGE-specific silencing and compensation [63,64,65]. In the pCAR1 system, the plasmid-encoded NAP Pmr interacts with chromosomal H-NS paralogues TurA and TurB, and plasmid acquisition triggers broader reprogramming of the recipient transcriptome rather than simple restoration of xenogeneic silencing [66,67].

Beyond preventing the constitutive expression of foreign DNA, xenogeneic silencing creates a regulatory reservoir of horizontally acquired genes whose expression can be selectively unlocked in response to specific environmental cues. This reversible control allows bacteria to exploit newly acquired functions only when they enhance fitness or pathogenicity. Silencing can be relieved either by directly interfering with NAP-DNA interactions or by altering the regulatory conditions that favor silencing. The expression of virulence genes subjected to NAP-mediated xenogeneic silencing is therefore mainly restored through two broad classes of mechanisms: (1) anti-silencing (counter-silencing) proteins, H-NS-related or not, that displace or remodel H-NS and related xenogeneic silencers, and (2) environmental or host-derived signals that modify promoter activity, DNA topology, or the abundance and stability of the silencing proteins. Interestingly, despite the diversity of environmental and host conditions encountered during infection, similar regulatory principles recur and will be revisited throughout the different stages of the infection cycle, as further illustrated in the following sections.

Finally, post-translational modifications of NAPs have gained significant attention over the last decade, particularly regarding their involvement in the regulation of xenogeneic silencing (Table 2) [68,69]. For example, temperature-dependent phosphorylation of H-NS at Ser42 is critical for silencing the MGE-acquired cold-inducible genes in the CP4So prophage of *Shewanella oneidensis* [70]. H-NS acetylation can also assist its displacement from the T3/T6SS clusters by EnR encoded in the *Edwardsiella* pathogenicity island 1 [71,72].

### 2.2. Adaptation to the Host Environment

Throughout their life cycle, pathogens are continuously exposed to physicochemical fluctuations such as temperature, pH, osmolarity, oxidants, and nutrient availability, both in environmental habitats and during infection. These signals are primarily sensed through dedicated regulatory systems, including two-component systems, alternative sigma factors, and the stringent response, which reprogram gene expression in response to specific cues [73,74,75,76]. NAPs provide an additional layer of adaptation by coupling changes in the physicochemical state of the cell to chromosome organization and transcription. Because their DNA-binding and architectural properties are sensitive to environmental conditions, NAPs can rapidly remodel nucleoid structure and alter gene expression, complementing dedicated signaling pathways during transitions between variable environments and host niches.

#### 2.2.1. Temperature

Temperature is probably the primary signal encountered by pathogens during the environment-to-host transition, with a difference that usually corresponds to 10–15 °C [77]. Temperature strongly influences DNA topology through changes in superhelical density, intrinsic DNA bending, particularly at AT-rich sequences, and duplex stability, thereby affecting DNA transactions [78,79,80]. H-NS has emerged as a key reader of temperature-dependent changes in DNA architecture, allowing bacteria to anticipate host-associated conditions. H-NS can sense temperature-induced structural transitions on DNA, as DNA bending below 32 °C promotes H-NS-mediated remodeling of the *virF* promoter in cooperation with Fis [81,82]. H-NS itself also displays thermosensitive properties, as increased temperature reduces nucleoprotein filament formation and DNA binding [83,84,85,86]. Therefore, temperature can relieve xenogeneic silencing by modulating H-NS binding to DNA. For example, the expression of plasmid-encoded virulence gene hemolysin in *E. coli* is strongly dependent on temperature-mediated changes in DNA flexibility that destabilize H-NS binding on the *hly* regulatory sequences [87]. In *S. flexneri*, the virulence genes carried on the horizontally acquired pINV plasmid are silenced by H-NS at temperatures ≤30 °C but become derepressed at host temperature (37 °C) through the anti-silencer VirB, which locally remodels DNA topology and antagonizes H-NS repression [88,89]. Conversely, H-NS also contributes to adaptation to colder environments by regulating cold-shock responses through changes in its oligomerization state and by increasing *hns* expression following temperature downshift [85,90,91]. Other NAPs contribute primarily to maintaining chromosome homeostasis during thermal fluctuations. HU buffers temperature-dependent changes in DNA supercoiling and supports transcriptional adaptation during cold stress, whereas Fis and Dps contribute to nucleoid organization and protection upon temperature changes [92,93,94,95].

#### 2.2.2. Acidic Environment

Before host colonization can occur, enteric pathogens must overcome the gastric barrier, another major physicochemical constraint encountered during the transition from environmental reservoirs to the host [96]. NAPs contribute to this early stage of infection by promoting stress tolerance through both regulatory and structural mechanisms. In *E. coli*, H-NS controls the activation threshold of the glutamate decarboxylase-dependent acid resistance system by directly binding the *gadA* and *gadX* promoters and repressing their expression in vitro and in vivo. This repression predominates during exponential growth and is relieved as σS accumulates during stationary phase, linking acid resistance primarily to physiological state rather than to a specific host-derived signal [97,98]. Other NAPs act on the parallel decarboxylase systems, HU being required for arginine-dependent acid resistance and IHF for the acid induction of the arginine and lysine pathways [99,100]. The same regulation operates in a pathogen: in *S. flexneri*, the plasmid-encoded H-NS paralogue Sfh represses *gadA* and *gadB*, so that carriage of pSf-R27 lowers survival at pH 2.5 [101]. In contrast, Dps provides a more direct layer of protection by shielding the chromosome from acid-induced damage. *dps* mutants of *E. coli* O157:H7 display reduced viability under acid stress [102,103], consistent with the pH-dependent modulation of Dps-DNA interactions that favors nucleoid protection under acidic conditions.

#### 2.2.3. Oxygen Limitation

Another environmental cue encountered by pathogens in both environmental reservoirs and host niches is oxygen availability. Oxygen limitation is a key determinant of bacterial physiology and pathogenicity, as many infection sites display heterogeneous and dynamic oxygen gradients, ranging from oxygen-rich surfaces to hypoxic or anaerobic microenvironments within tissues [104,105]. However, compared with other environmental signals, the contribution of NAPs to oxygen adaptation remains relatively unexplored. Nevertheless, several studies suggest that chromosome architecture participates in the transcriptional response to this stress. For example, in *Salmonella enterica*, Fis expression is sustained during anaerobic growth, and its promoter functions as a topological switch that responds to changes in DNA supercoiling under anaerobic conditions [106]. In *E. coli*, the HU regulon includes genes involved in both aerobic and anaerobic respiration, suggesting that HU contributes to the adaptation of energy metabolism during oxygen limitation [107]. The strongest evidence comes from mycobacteria, where Lsr2 contributes to the hypoxic response by regulating numerous oxygen-responsive genes, including the dormancy-associated DosS/DosR two-component system, thereby coupling chromosome organization to the establishment of hypoxia-induced persistence [108,109].

#### 2.2.4. Nutrient Limitation

Beyond abrupt physicochemical stresses, pathogens frequently experience prolonged nutrient limitation while persisting in environmental reservoirs, biofilms or intermediate hosts before encountering a mammalian host. Starvation is characterized by a switch to stationary phase that leads to a drastic reduction in growth rate [110]. The transition to stationary phase is principally driven by the stringent response, in which nutrient limitation triggers (p)ppGpp production and broad transcriptional reprogramming [111]. This transcriptional reprogramming also reshapes the NAP repertoire. Because the *fis* promoter is highly sensitive to NTP pools and ppGpp, nutrient limitation rapidly depletes Fis, whereas σS-dependent induction of *dps* makes Dps the dominant protein of the stationary-phase nucleoid [26]. This reciprocal replacement of Fis by Dps establishes a distinct chromosomal state adapted to long-term persistence rather than representing a simple consequence of growth arrest. Rather than simply compacting the chromosome, Dps promotes a protective nucleoid architecture while preserving its functional organization. Indeed, Dps-mediated protection does not necessarily result in transcriptional silencing, as Dps can protect DNA while preserving RNA polymerase access and chromosome dynamics [112,113]. Maintaining a protected yet transcriptionally competent chromosome may therefore be particularly advantageous for environmental pathogens, allowing rapid physiological reactivation upon encountering host-associated conditions. Other NAPs, including IHF and Fis, also link nutrient availability to starvation adaptation and virulence-associated programs [114,115,116].

## 3. Establishing Infection: Colonization, Adhesion and Invasion

Colonization is the establishment of bacterial populations within the host and represents a critical step in infection [117]. For extracellular pathogens, colonization involves finding a niche to proliferate and form biofilms, whereas intracellular bacteria must find an entry route to invade their target cells. Besides the abiotic stresses discussed above, bacteria must face multiple challenges to achieve efficient colonization, including competition with the local microbiota, motility to navigate environmental cues, and biofilm formation.

### 3.1. Competition with Microbiota

To colonize a host, pathogenic bacteria must compete with commensal communities to access nutrients and ecological niches [118]. This is particularly crucial in environments with a dense and established microbiota, such as the gastric niche. Pathogens can gain a competitive advantage by delivering antibacterial toxins to neighboring bacteria through specialized secretion systems. These systems are among the most tightly regulated virulence-associated machineries in pathogenic bacteria, and nucleoid-associated proteins (NAPs) play a central role in controlling their conditional expression (see Table 3). Across diverse pathogens, NAPs impose basal transcriptional silencing on secretion loci, ensuring that these energetically costly systems remain off under non-inducing conditions. Virulence activation occurs only when counter-silencers or host-responsive regulators overcome this repression [27]. The type VI secretion system (T6SS) has been described as essential for colonization in numerous pathogenic species in the gastrointestinal tract. H-NS is a well-described repressor of T6SS in several bacterial pathogens, including *S. Typhimurium*, *Klebsiella pneumoniae*, and *Vibrio parahaemolyticus* [119,120,121]. In *S. Typhimurium*, SPI-6 (Salmonella Pathogenicity Island 6) encodes a T6SS and associated proteins Hcp-1, Hcp-2 and Tae4, an antibacterial effector. Under non-inducing conditions, H-NS binds the divergent promoter region of SPI-6 and oligomerizes along the DNA, extending over a large region of the locus to establish xenogeneic transcriptional silencing [120]. In this context, counter-silencing may occur in response to environmental cues. In the intestinal environment, bile salts can induce T6SS expression in *S. Typhimurium*, as reported for *V. cholerae*, suggesting that spatial variations in these host-associated signals can contribute to restricting T6SS activity to specific niches [122]. This conditional expression is ecologically relevant because T6SS-mediated antibacterial activity can shape the composition of competing bacterial communities. In *S. Typhimurium*, SPI-6 contributes to the killing of *Klebsiella oxytoca* and *Klebsiella variicola*, but not *E. coli* [122], whereas in *Helicobacter hepaticus*, T6SS activity can instead promote stable interactions with members of the resident microbiota [123].

H-NS is not the only NAP involved in the regulation of interbacterial competition. In the opportunistic pathogen *Enterobacter cloacae*, HU binds promoter regions of T6SS-1 and T6SS-2 to activate their expression, while deletion of both HU subunits impairs bactericidal activity against *E. coli* [124]. Similarly, IHF binds and transactivates the VflT6SS2 cluster in the emerging foodborne pathogen *Vibrio fluvialis*. Mutations of the genes encoding IHF subunits in this species lead to reduced expression and secretion of associated Hcp [125].

Bacteria can also interfere with competitors through non-biocidal mechanisms, including inhibition of biofilm formation. Although no direct link has been established between NAPs and anti-biofilm activity, H-NS influences the expression of capsule-associated genes in *K. pneumoniae* (see Section 4.3.1) and represses the *eps* operon encoding exopolysaccharides required for plant colonization by *Dickeya dadantii* [126,127,128].

### 3.2. Motility and Chemotaxis

Motility is essential for bacterial survival and host colonization, allowing cells to explore their environment, locate favorable niches, and escape unfavorable conditions. Bacteria move across liquid and solid media through distinct mechanisms, including swimming, twitching or gliding, which rely primarily on flagella and/or type IV pili (T4P) [129,130,131]. In many pathogens, motility must be coordinated with the transition to a sessile lifestyle, as cells switch from active exploration to attachment and biofilm formation [130]. 

NAPs contribute to this plasticity by regulating the expression of genes involved in flagellar and pilus biosynthesis or chemotaxis. Fis activates flagellar genes in *S. enterica*, and *fis* mutants exhibit reduced motility [132]. Similarly, in the phytopathogen *Dickeya* spp., *ihf* mutants show impaired motility by losing their ability to swim and twitch [130,133]. This phenotype results from the IHF-dependent activation of *fliZ*, encoding a regulator involved in the transition between motility and biofilm formation, and the *pil* genes, encoding T4P components [130]. NAPs can also integrate motility regulation with broader transcriptional programs through alternative sigma factors. In *V. cholerae*, H-NS stabilizes *rpoS* mRNA, although the underlying mechanism remains unclear [134,135]. RpoS promotes the expression of several genes involved in flagellar function and chemotaxis, including *flaA*, *flaC* and *cheR-2*, while H-NS directly represses *flrA*, a master regulator of flagellar genes. This repression is relieved in the presence of RpoS, which also activates *ihf*, whose product can counter-silence H-NS-repressed promoters by displacing H-NS from DNA [136].

To find suitable niches, bacteria use chemotaxis to follow environmental cues. In *V. cholerae*, transcriptomic analysis of an *hns* mutant revealed that H-NS positively regulates numerous chemotaxis genes, including those encoding methyl-accepting chemotaxis proteins (MCPs), which sense environmental signals and relay them to the flagellar motor. Consistent with these transcriptional changes, the *hns* mutant showed reduced motility and impaired attraction to serine and glycine [136,137], as previously reported in an El Tor biotype *hns* mutant [135]. Interestingly, loss of chemotaxis can, under some conditions, favor colonization, potentially by promoting less directed exploration and allowing bacteria to access and establish themselves in otherwise unexplored niches [138]. However, the pleiotropic effects of *hns* deletion, including reduced fitness, may counteract this potential advantage and contribute to the impaired intestinal colonization observed in mice [139].

### 3.3. Biofilm

Biofilm formation is a key determinant of host colonization, persistence, and chronic infection across diverse bacterial pathogens [140]. NAPs contribute to this process through distinct but complementary mechanisms that link intracellular regulation to cell-surface and extracellular matrix organization.

Lsr2 can couple intracellular signaling to biofilm-associated pathogenicity: cyclic di-GMP binds Lsr2 at a 1:1 stoichiometry and promotes activation of *hadD*, a dehydratase required for keto-mycolic acid synthesis [141]. By remodeling mycolic acid composition and strengthening the mycobacterial envelope, this pathway promotes biofilm formation, illustrating how Lsr2 can connect second messenger signaling to cell-surface remodeling and persistence. IHF can additionally promote biofilm formation through transcriptional regulation of surface-associated factors. In *K. pneumoniae*, deletion of either *ihfA* or *ihfB* strongly impairs biofilm formation. Transcriptomic and qRT-PCR analyses showed that IHF positively regulates genes associated with capsular polysaccharides, LPS, cellulose, and type I and III fimbriae, linking IHF-dependent transcriptional regulation to multiple determinants of adhesion and biofilm formation [142].

Remarkably, NAPs can also act beyond the nucleoid, with some being secreted outside the bacterial cell and contributing directly to the extracellular architecture of biofilms. In several pathogenic biofilms, the DNABII proteins HU and IHF are found within the extracellular matrix, where they bind extracellular DNA (eDNA), stabilizing the extracellular polymeric substance (EPS) and thereby sustaining biofilm integrity [140,143]. Targeting these proteins with specific antibodies disrupts the eDNA scaffold and causes biofilm collapse, highlighting their structural contribution to biofilm integrity [140]. Recent structural and biophysical analyses further showed that HU can bridge eDNA and the bacterial envelope through interactions with lipopolysaccharide (LPS), thereby promoting bacterial aggregation and stable attachment within the biofilm matrix. Consistently, exogenous HU promotes bacterial incorporation into an eDNA-rich matrix, whereas HU depletion destabilizes biofilms [144].

### 3.4. Adhesion

Adhesion is a critical early step in infection, allowing bacteria to establish stable contact with host cells. It relies on numerous surface-associated virulence factors, including pili and fimbriae, whose pilins and adhesins mediate attachment to host surfaces [145]. Other systems can contribute to this process, including the type VI secretion system (T6SS) and its Hcp component, as reported in *E. coli* or *Campylobacter jejuni* [121,146,147]. Consistent with the involvement of these surface and secretion systems, mutations in NAP-coding genes can alter bacterial adhesion, either reducing it, as reported for Lsr2 in *Mycobacterium tuberculosis* and HU in *E. cloacae* [124,148] or increasing it, as observed for Fis in *K. pneumoniae* and H-NS in *A. baumannii* [149,150].

These phenotypes can result from direct or indirect regulation of adhesion-associated genes. H-NS and Fis provide particularly clear examples of this transcriptional control. In *V. parahaemolyticus*, H-NS binds promoter regions within the T6SS2 locus, repressing transcription of genes required for adhesion, including *hcp2*, whose product forms the translocon through which T6SS2 mediates this function [119,151]. Accordingly, *hns* deletion results in increased T6SS2 activity [119]. In *K. pneumoniae*, H-NS similarly binds and represses the *hcp* promoter, reducing T6SS activity and consequently the expression of type 1 pili required for adhesion and invasion of colonic epithelial cells [121]. Fis can also directly repress adhesion determinants in this species by binding the *mrkA* and *mrkH* promoters, encoding the major pilin and a regulator of type III pili, respectively [149].

A related mechanism operates in enteropathogenic and enterohemorrhagic *E. coli* (EPEC and EHEC), where NAPs regulate the locus of enterocyte effacement (LEE), a cluster of horizontally acquired virulence genes encoding the T3SS and its associated proteins. Reactivation of the locus is dependent on the LEE1-encoded master regulator Ler, an H-NS-like protein that disrupts the H-NS nucleoprotein structure by wrapping AT-rich DNA in non-cooperative toroidal complexes [152,153]. Ler-dependent transcription activation is also enhanced by the Pch protein, which reduces H-NS binding to the LEE1 promoter [154]. Members of the H-NST family, a family of truncated H-NS derivatives, also act as positive regulators of transcription by counteracting H-NS within LEE in a Ler-independent manner both in EHEC and EPEC [155,156,157]. In EPEC, Fis is required for the activation of the *ler* promoter, which controls the LEE pathogenicity island and thereby promotes production of the EspA, EspB and EspD proteins required for intimate attachment and the formation of attaching-and-effacing lesions in the intestinal epithelium [158].

NAPs can likewise control adhesion through regulatory switches that determine the expression state of pili. In enterotoxigenic *E. coli*, H-NS represses genes encoding the type 4 pilus Longus and surface antigen CS3, both involved in epithelial adhesion [159,160]. In contrast, H-NS is an activator of T3P gene expression in *K. pneumoniae*, by positively regulating *mrkA* [126]. In pathogenic *E. coli*, Fis regulates the invertible *fimS* promoter controlling type 1 pilus expression by promoting switching to, or maintaining it in, the OFF state, thereby reducing adhesion [161,162,163]. Fis can act through interaction with, or repression of FimE, one of the recombinases that controls this ON-OFF switch [161,162,163]. The other *fimS* switch recombinase, FimB, is transcriptionally regulated by the binding of NagC on two distant genomic elements. IHF acts as a cofactor for NagC by binding between the two operator sequences and inducing a DNA hairpin that brings them into proximity. This facilitates NagC binding to both operators, thereby activating *fimB* expression and promoting the ON orientation of *fimS*. Adhesin-coding genes can also be targeted by NAPs, such as *btaE* and *omp25d* in *Brucella* species that are repressed by MucR, an H-NS-like NAP [27]. This repression can be relieved by the counter-silencer MdrA in response to host-associated signals, linking adhesin expression to environmental conditions [164].

Once again, NAPs can also display functional versatility, with some contributing directly to bacterial adhesion after being secreted to the cell surface. In mycobacteria, HU is secreted to the cell surface, where it binds to glycosaminoglycans, including hyaluronic acid, on epithelial cells [165,166]. In *Mycobacterium bovis*, binding of Hlp (HU) to the A549 pneumocyte line is enhanced by collagen, a major constituent of the extracellular matrix [167]. Interestingly, this interaction can also be targeted by host cells. The histone methyltransferase SUV39H1 in THP-1 macrophages methylates HU, reducing its ability to promote biofilm formation and potentially limiting bacterial adhesion [168].

### 3.5. Invasion

Following attachment to host cells, invasive bacteria must overcome the cellular barrier and activate the virulence programs required for entry into epithelial or other target cells. This transition requires the precise deployment of secretion systems and their effectors, whose expression is tightly tuned to environmental signals. Through their control of virulence regulators and secretion system genes, NAPs integrate these cues to coordinate the transition from attachment to cellular invasion.

In *S. enterica*, SPI-1 invasion genes that drive the type III secretion system and its associated effectors are tightly controlled by NAPs. This provides a clear example of xenogeneic silencing, whereby H-NS keeps the horizontally acquired SPI-1 locus transcriptionally silent under non-invasive conditions, while activation of SPI-1 remodels H-NS occupancy, reshapes the local chromatin landscape, and enables Hil-D-driven transcription of invasion genes [48,169]. HU also promotes invasion by upregulating *hilA*, *hilC* and *hilD* [170]. Accordingly, *hupA* and *hupB* mutations reduce expression of these genes, leading to a defect in invasion of CHO epithelial cells. IHF is also involved in this regulatory network: IHF represses *hu* genes, whereas HU activates *ihf* expression, and SPI-1 genes are upregulated in an *ihfAB* double mutant [116,170]. This regulatory interplay may therefore contribute to tuning SPI-1 expression and, consequently, the invasive phenotype.

The NAP-dependent control of invasion can be particularly striking when it acts as an environmental switch. In *Shigella*, invasion is mediated by a plasmid encoding the Mxi-Spa type III secretion system and its Ipa invasins, whose expression is controlled by the VirF/VirB regulatory cascade [171]. At temperatures below 32 °C, H-NS binds the *virB* and *virF* promoters and represses their expression [172,173,174]. Upon entry into the host and exposure to permissive temperatures, this repression is relieved, potentially through temperature-dependent changes in DNA curvature [81] and competition between H-NS and the activating NAPs IHF and Fis at these promoters [175,176].

NAPs can also influence the transition from a non-invasive or commensal state to pathogenic behavior. In the K-12 commensal *E. coli* strain, a gain-of-function mutation of the HUα subunit is sufficient to confer an invasive phenotype. Given the pleiotropic effects of NAPs, this phenotype could reflect the combined deregulation of multiple pathways. However, the mutation is also associated with constitutive activation of curli fibers, extracellular amyloid fibers involved in biofilm formation and invasion, normally kept quiescent in this strain [177]. Likewise, in the oral bacterium *P. gingivalis*, which can act as an opportunistic pathogen, Huβ positively regulates the K-antigen capsule, a major virulence factor involved in aggregation, dissemination and invasion [178,179]. 

Taken together, these examples illustrate several recurring mechanisms by which NAPs contribute to host colonization, adhesion and invasion across diverse bacterial pathogens. First, NAPs frequently act as xenogeneic silencers to prevent premature expression of horizontally acquired virulence functions, which can subsequently be relieved by counter-silencers or host-associated signals. Second, changes in environmental conditions can modify DNA topology and NAP-DNA interactions, providing a rapid mechanism to couple environmental sensing to transcriptional reprogramming. Third, NAPs often coordinate the expression of multiple surface-associated and secretion-associated determinants, thereby linking chromosome organization to broader transitions between motility, adhesion, biofilm formation and invasion. Although the specific NAPs and regulatory targets differ between species, these recurring principles highlight a common role for NAPs as regulatory hubs that integrate environmental cues with the coordinated regulation of virulence-associated functions.

## 4. Surviving Host Defenses

After breaching host barriers and establishing infection, pathogens must withstand a wide range of host defenses. This marks a new stage of infection, during which NAPs help bacteria adapt to, withstand, and modulate these host-induced stresses.

### 4.1. Non-Immune Cells

Non-immune cells are an important first line of defense against pathogens, recognizing bacterial components through membrane Toll-like receptors (TLRs) and producing cytokines that attract and activate immune cells. NAPs can modulate this response either indirectly, through regulation of bacterial surface components, or directly through interactions with host or extracellular molecules.

Antimicrobial peptides (AMPs) are small peptides secreted by epithelial and professional immune cells to target bacteria and promote bacterial killing through direct bactericidal activity, repression of biofilm formation or stimulation of immune responses [180]. Several bacterial defenses against AMPs are under NAP control. The clearest example is the Sap (sensitive to antimicrobial peptides) transporter, which imports AMPs into the cytoplasm for degradation [181]. First described in *S. Typhimurium*, the *sapABCDF* operon is directly activated by Fis in the phytopathogen *D. dadantii*, where Fis binds the regulatory regions of the locus [182,183]. NAPs can also act indirectly, through regulators controlling outer membrane porins. In *V. cholerae*, resistance to the peptide P2 is mediated mainly by OmpU, whose expression is activated by ToxR, the master regulator of the ToxR virulence cascade [184], whereas *ompT* is repressed by both ToxR and H-NS [139]. *ompU* itself is an H-NS target in *V. fischeri* but not in *V. cholerae.* OmpT contributes to AMP resistance in EHEC, EPEC and UPEC through OmpT-like proteases that cleave and inactivate LL-37 [185,186], while outer-membrane vesicles enriched in the matrix protein Bap1 can sequester LL-37 rather than degrade it [187]. Although a StpA binding site has recently been identified in the *E. coli ompT* promoter, functional NAP-dependent regulation of OmpT has yet to be demonstrated. This example also highlights the difficulty of linking NAP activity to a specific regulatory pathway, given their extensive network of direct and indirect effects.

Beyond AMP secretion, host cells can also restrict infection by triggering programmed cell death, thereby disrupting the cellular niche required for bacterial survival [177,188]. This is illustrated by a gain-of-function *hu* mutant in invasive *E. coli* strains, which prevents intestinal cell death by inactivating two major pro-apoptotic proteins, Bim and Puma [177]. A related mechanism occurs in *M. tuberculosis*, where the NAP EspR, delivered into macrophages through the ESX-1 system, limits apoptosis by interfering with MyD88-dependent signaling and reducing caspase-3 and caspase-8 activation [189].

In some *Vibrio* species, H-NS controls virulence programs that directly target non-immune epithelial cells. In *V. cholerae*, the cholera toxin operon *ctxAB* is located in the lysogenic filamentous prophage CTXφ. H-NS binds directly to multiple sites across the *ctxAB* promoter and silences it by default, keeping toxin production off outside the host. During infection, the AraC-type activator ToxT, the terminal regulator of the ToxR virulence cascade, displaces H-NS from overlapping binding sites to switch on *ctxAB* transcription [190,191]. H-NS also directly silences the *tcp* operons, encoding the toxin-coregulated pilus, which, together with cholera toxin, is essential for intestinal colonization and intoxication [190,192]. Similarly, in *Vibrio vulnificus*, H-NS represses *rtxA1*, which encodes the MARTX toxin, until counter-silencing by HlyU relieves this repression [193,194]. Once expressed, the MARTX effector repertoire induces apoptosis and necrosis, but also disrupts epithelial and endothelial barriers [194,195]. A similar mechanism operates in *V. parahaemolyticus*, where H-NS binds promoter-proximal regions within the Vp-PAI island and represses the regulator *vtrA*, the T3SS2 apparatus gene *vopB2* and the thermostable direct haemolysin (TDH) gene *tdh2* [119]. Loss of H-NS increases haemolytic activity and enterotoxicity, consistent with the respective contributions of TDH and T3SS2 to these activities.

As discussed earlier, capsules and membrane proteins are essential determinants of host-bacteria interactions and are also involved in TLR recognition and immune activation. Among HU-regulated capsule genes, *epsC* is necessary to limit IL-6 and IL-8 production by fibroblasts following infection by *P. gingivalis* [178,179,196]. In *Aggregatibacter actinomycetemcomitans*, IL-1β is internalized by viable biofilm cells and accumulates at the periphery of the nucleoid, where it interacts with HU. Whether this sequestration dampens the inflammatory response remains to be established. In *K. pneumoniae*, the H-NS-regulated capsule locus limits interaction with TLR-2 and TLR-4, therefore reducing IL-8 secretion [126,197].

In adherent-invasive *E. coli* (AIEC), sialic acid sensing provides a NAP-regulated route to immune escape. Sialic acids are host-derived nutrients whose uptake requires the outer-membrane channel NanC, which is repressed by Fis. Accordingly, reduced Fis levels in the AIEC strain LF82 increase sialic acid uptake [163]. Once internalized, sialic acid inactivates NanR and NagC, reducing *fimB* expression and thereby limiting FimB-mediated switching of *fimS* to the ON state [198]. IHF also contributes to *fimB* regulation, linking this response to the NAP-dependent control of the fimbrial switch [198]. The resulting repression of type 1 fimbriae, which are pro-inflammatory, dampens the host response [195]. As sialic acid levels increase during inflammation, this system may therefore allow *E. coli* to sense host defense activation and pre-emptively reduce a pro-inflammatory adhesin.

Extracellular NAPs can also modulate host inflammatory responses through direct interactions with host or extracellular molecules. In mycobacteria, the HU-like proteins Hlp and MDP1 bind eDNA and stimulate TLR-9 activation in spleen and alveolar cells, leading to the production of IL-8, γ-interferon and immunoglobulin G [199,200,201]. Consistent with this immunomodulatory role, down-regulation of MDP1 in *M. bovis* reduces IL-1β, IL-10 and interferon-γ levels [202]. In *H. pylori*, NapA, a secreted Dps-family NAP, also contributes to the pro-inflammatory response [203].

### 4.2. Neutrophils and NETosis

Neutrophils are a first line of host defense during inflammation. They can eliminate pathogens either intracellularly through phagocytosis and reactive oxygen species (ROS) production or extracellularly through the formation of neutrophil extracellular traps (NETs). Some pathogens nevertheless promote neutrophil recruitment, exploiting the tissue damage that accompanies their migration. They must therefore withstand the antimicrobial mechanisms they have triggered, and several NAP-dependent strategies contribute to this process, notably through secretion and extracellular activities.

*H. pylori* is one of these neutrophil-recruiting species. Upon its release into the extracellular space, NapA crosses the endothelial barrier *via* intracellular transport and stimulates adhesion and transmigration of neutrophils through the endothelium [204,205,206]. Although initially characterized as a “neutrophil-activating protein”, its ability to induce chemokine release remains controversial. NapA, a Dps-like protein, also stimulates the neutrophil oxidative burst, potentially contributing to tissue damage that may favor colonization [204,207,208,209,210]. *H. pylori* must therefore withstand the resulting oxidative stress through detoxifying enzymes such as catalase [211]. Although these enzymes are NAP-regulated in other species, such control has not been described for conventional NAPs in *H. pylori*. However, the histone-like protein HP119 regulates antioxidant genes such as *sodB* and *trxR*, and its deletion also reduces *napA* expression [212].

Another non-phagocytic way of bacterial killing is through the formation and release of NETs, composed of DNA associated with antimicrobial agents such as myeloperoxidase or histones and can trap bacteria and impair biofilm growth [213,214]. Multiple NAPs directly contribute to bacterial defense against NETs. Extracellular HU can bind both bacterial eDNA and the host DNA forming NETs. By promoting a B-to-Z transition in DNA, HU reduces the susceptibility of bacterial eDNA to degradation while also altering the conformation of adjacent NET DNA. This conformational change causes the detachment of associated antimicrobial proteins, reducing their contact with trapped bacteria [215]. H-NS provides a complementary mechanism: it binds the remaining B-form DNA within NETs, inducing its condensation and retraction and thereby preventing the formation of new NET structures and reducing the access of antimicrobial proteins to bacteria [214].

### 4.3. Macrophage

#### 4.3.1. Macrophage Recognition and Internalization

For bacteria interacting with macrophages, phagocytic uptake can represent either an obstacle to avoid or a step toward an intracellular niche for survival and replication. NAPs contribute to this balance by regulating bacterial surface properties and adhesion proteins involved in recognition and internalization. In *M. tuberculosis*, deletion of *hupB* reduces bacterial entry into murine RAW264.7 macrophages [216]. Similarly, treatment with aptamers targeting HU reduces *M. tuberculosis* invasion of human THP-1 macrophages by approximately half. Because these aptamers bind surface-associated HU, the reduced uptake was proposed to result from an inhibition of its extracellular adhesin function [217]. Consistent with this interpretation, the mycobacterial HU homolog Hlp is exposed to the bacterial surface and binds to collagen, promoting adhesion of *M. bovis* to human U937 macrophages [167] (Section 3.4). Consequently, although no direct regulatory mechanism has been demonstrated to date, several studies support a role for HU in the internalization of mycobacterial species by macrophages. The mechanisms underlying these observations remain to be elucidated.

Conversely, NAPs can limit macrophage uptake by controlling surface structures that shield bacteria from phagocytic recognition. In *K. pneumoniae*, H-NS represses the *cps* locus and its regulator *rcsA*, thereby limiting capsule production [126]. Accordingly, *hns* mutants display increased capsule biosynthesis and a hypermucoviscous phenotype, resulting in a more than 50-fold reduction in internalization by murine and human macrophages. Importantly, this regulation is temperature dependent: H-NS-mediated repression of the capsule locus is 4-fold stronger at 25 °C than at 37 °C, suggesting that the temperature shift encountered upon entering the host contributes to derepression of capsular genes and increased surface shielding [129].

This regulatory balance can also be influenced by the host. Cholesterol-rich lipid rafts in the macrophage membrane induce an H-NS-dependent signaling pathway that leads to the repression of the capsular genes *galF*, *wzi*, and *manC*, as well as genes encoding LPS and other membrane proteins such as *rfaD*, *ompA*, *ompK35*, and *ompK36* [218]. The resulting reduction in surface shielding promotes bacterial uptake and illustrates how host-mediated signals can modulate NAP transcriptional activity, altering the outcome of bacteria-macrophage interaction.

Beyond the passive shielding provided by surface structures, NAPs can also regulate active mechanisms that block phagocytic uptake. In *Pseudomonas aeruginosa*, the H-NS family proteins MvaT and MvaU bind the Vfr-dependent P*exsA* promoter and restrict production of ExsA, the master activator of the type III secretion system [219]. Loss of either NAP derepresses P*exsA*, elevates ExsA levels and enhances secretion of T3SS substrates. Among the translocated effectors, ExoS and ExoT inactivate Rho-family GTPases and thereby interfere with phagocytic uptake [220], so that NAP-mediated silencing of a single promoter ultimately sets the extent to which the bacterium resists internalization by macrophages.

#### 4.3.2. Within the Phagosome

Once inside macrophages, bacteria encounter a wide range of stresses that threaten their survival. Among these, iron limitation, oxidative stress, and acidic stress are particularly characteristic of the phagosomal environment.

##### Iron Acquisition

Iron availability is tightly controlled within the host, forcing intracellular pathogens to activate iron acquisition systems to overcome this limitation. In *M. tuberculosis*, the NAP HupB contributes directly to this response by promoting the biosynthesis of the mycobactin and carboxymycobactin siderophores under iron-limiting conditions [216]. HupB binds an AT-rich region within the *mbtB* promoter and directly activates the *mbt* siderophore biosynthetic locus. This regulation is coordinated with the iron-responsive regulator IdeR, which represses both *hupB* and the *mbt* locus when iron is abundant [216,221]. Iron availability therefore controls siderophore production at two levels: by regulating HupB abundance and by determining whether HupB can activate the *mbt* locus. Accordingly, loss of HupB reduces *mbt* expression and siderophore production under iron limitation, compromising bacterial survival in macrophages [216].

##### Protection Against DNA Damage

Following phagocytosis, macrophages expose intracellular bacteria to a range of antimicrobial stresses, including reactive oxygen and nitrogen species (ROS and RNS) generated during the oxidative burst [222]. These reactive species, together with iron-mediated Fenton chemistry, can damage bacterial macromolecules, particularly DNA. NAPs contribute to bacterial protection against this damage through complementary mechanisms, including direct shielding of the chromosome, control of iron homeostasis, regulation of detoxifying enzymes, and promotion of DNA repair.

In vitro studies have shown that some NAPs are able to bind DNA to protect it from degradation caused by H_2_O_2_. Dps was among the first NAPs identified as a DNA-protective factor: its production is correlated with resistance to hydrogen peroxide, while *dps* mutants show increased sensitivity to oxidative stress [223,224]. Dps binds DNA in vitro and in vivo and prevents its degradation by H_2_O_2_, supporting a direct physical role in DNA protection [224]. Other NAPs have also been linked to this process, including Hup and NapA in *H. pylori* [209,225] and Lsr2 in *M. tuberculosis* [222]. In *M. tuberculosis*, the genomic regions covered by Lsr2 do not correspond to genes involved in ROS regulation, suggesting that Lsr2 could act as a physical barrier against damaging agents, rather than through transcriptional control of genes encoding detoxifying enzymes [222]. Consistent with a protective role for these proteins, deletion of *hupB* in *M. tuberculosis* reduces resistance to oxidative stress, while loss of H-NS in *A. baumannii* and Lsr2 in *Mycobacterium abscessus* increases sensitivity to DNA-damaging agents, including mitomycin C and H_2_O_2_ [226,227,228]. Interestingly, *lsr2* mutants in *M. tuberculosis* do not show increased sensitivity to these agents [148], suggesting that other DNA-protective mechanisms may compensate for Lsr2 loss.

NAP-mediated DNA protection is also linked to iron homeostasis, as excess labile iron promotes oxidative damage through the Fenton reaction. In *V. cholerae*, H-NS positively regulates the expression of the bacterioferritin gene *bfr*, which contributes to iron sequestration [139]. Some NAPs can also bind and sequester iron directly. Dps-family proteins, including the Bacillus subtilis orthologue MrgA, are ferritin-like proteins that oxidize Fe^2+^ to Fe^3+^ and store iron within their protein shell [229,230,231]. By limiting the pool of reactive Fe^2+^, Dps reduces Fenton chemistry and thereby protects DNA from oxidative damage [229,232]. Mycobacterial MDP1 exhibits similar ferroxidase and iron storage activities despite being unrelated to the ferritin superfamily, suggesting a similar role in limiting iron-mediated oxidative damage [233]. In *Francisella tularensis*, HU similarly binds and protects DNA from Fenton-derived damage, contributing to intracellular growth and full virulence [234].

NAPs can themselves be regulated according to iron availability. In *H. pylori*, NapA possesses iron-binding activity and is induced under low-iron conditions, while its expression is negatively regulated by Fur, the major regulator of iron uptake and storage [209,235,236]. Extracellular or secreted NAPs may additionally influence host iron homeostasis: in *Brucella abortus*, Dps delivered into the host cytosol transiently suppresses the labile iron pool early in infection, while infection with a *dps* mutant induces the ferritin subunits FTH1 and FTL1, ultimately affecting ferritinophagy and macrophage susceptibility to ferroptosis [237].

Beyond direct DNA protection and iron sequestration, NAPs can regulate antioxidant defenses. In *S. Typhimurium*, Fis regulates *sodA*, *sod B* and *sodC* encoding superoxide dismutases that convert reactive superoxide anions into less damaging hydrogen peroxide [132]. In *F. tularensis*, HU is also a positive regulator of SodB and redox-protective proteins, such as glutaredoxin and thioredoxin [234]. Fis also activates the expression of *grxA*, *katG* and *ahpC* in *Yersinia pseudotuberculosis* [238]. Conversely, H-NS represses *sodC*, *katB* and *katG* in *V. cholerae* under non-stress conditions, illustrating how NAPs can tune antioxidant capacity according to environmental needs [135,139].

Finally, protection from reactive species cannot prevent all DNA damage, and bacteria must repair lesions that escape these mechanisms. Although NAPs are well known to participate in DNA repair, their contribution in an infectious context remains less characterized. In *H. pylori*, HU was identified in a screen for mutants defective in DNA repair, a process important for persistent colonization [225]. HU contributes to recombinational repair, and the *hupB* mutant is highly sensitive to oxidative stress and less able to survive within macrophages, suggesting that HU-mediated DNA repair may contribute to intracellular persistence.

##### Response to Phagosome Acidification 

pH is an important stress factor not only in the environment, but also in the intracellular niche. Once inside macrophages, bacteria must cope with progressive acidification of the phagosome, with pH decreasing from approximately 6.4 to 5.2 in activated macrophages and below 5.0 in phagolysosomes [202]. NAPs contribute to this adaptation by sensing or responding to changes in pH and modifying chromosome organization and gene expression (see also Section 2.2.2).

In *E. coli*, low pH directly affects nucleoid organization through HU. At pH 5, nucleoids become less condensed than at pH 7, forming rigid filaments rather than compact DNA bundles. This transition depends on the formation of HUαα multimers and is accompanied by increased HU binding, resulting in changes in the expression of numerous genes, including increased *dps* expression [239]. Consistent with this role, HU homologs are also required for growth under acidic conditions in intracellular pathogens: deletion of *hup* in *H. pylori* and downregulation of *hlp* in *M. bovis* cause growth defects at pH 5.3 and below [202,225].

In *Brucella melitensis*, MucR links adaptation to the acidic intracellular environment with the regulation of virulence functions. The *16MΔmucR* mutant displays reduced resistance to low pH [240], potentially reflecting impaired activation of BvrR, a component of the BvrR/BvrS two-component system that senses acidic host-associated conditions and regulates the corresponding stress-response pathways [164,241].

In *S. Typhimurium*, activation of the pH-sensing two-component system PhoP/PhoQ is critical for survival in the acidic macrophage environment [242]. PhoP regulates gene expression through distinct mechanisms: whereas core genes are directly controlled by PhoP, horizontally acquired genes are regulated through PhoP-mediated counter-silencing of H-NS. Acidification is first sensed by PhoQ, which generates phosphorylated PhoP that activates the horizontally acquired regulator *ssrB*. SsrB in turn displaces H-NS from the *ugtL* promoter, and UgtL promotes PhoQ autophosphorylation, thereby reinforcing PhoP activation in a positive feedback loop. This loop is further amplified during macrophage infection by Lon-dependent degradation of H-NS, which relieves silencing of horizontally acquired loci [243,244]. Functionally, this cascade sustains a low-pH-inducible, PhoP/PhoQ-dependent acid tolerance response that protects bacteria against inorganic acid stress, allowing phagosomal acidification to act as a signal for adaptation.

Acidification of the phagosome also triggers expression of the SPI-2 T3SS, which is essential for intracellular adaptation [245,246]. Under non-inducing conditions, H-NS, Hha, and YdgT occupy SPI-2 promoters and impose basal repression, whereas acidic intraphagosomal cues activate SsrB, which binds these promoters and counter-silences H-NS, enabling transcription of the T3SS and its effectors [4,245,247]. IHF and Fis further facilitate promoter accessibility, highlighting the multilayered NAP control of SPI-2 expression [4]. The resulting T3SS delivers effectors that remodel the phagosomal vacuole, drive endosomal tubulation, and redirect host trafficking pathways, thereby supporting intracellular replication during macrophage infection [245,248].

##### Phagosomal Escape

For pathogens that escape the phagosome, access to the host cytosol requires disruption or remodeling of the phagosomal compartment. In *B. abortus*, intracellular bacteria reside initially in endosomal Brucella-containing vacuoles (BCVs), which progressively acquire endoplasmic reticulum (ER) membranes to form ER-derived replicative organelles. This process depends on the VirB type IV secretion system (T4SS), as *virB* mutants fail to sustain interactions with the ER and ultimately undergo lysosomal degradation [249]. Expression of this intracellular survival machinery is controlled by MucR. MucR preferentially binds extended AT-rich DNA regions and oligomerizes to repress transcription, with genome-wide binding detected at hundreds of loci, including those encoding the VirB T4SS and its effectors [164]. MucR binding sites overlap with those of the transcriptional activators VjbR and BvrR, suggesting that host-associated signals may relieve MucR-mediated repression through a silencer/counter-silencer mechanism and thereby activate *virB* expression.

In *M. tuberculosis*, phagosomal escape is driven by the type VII secretion system ESX-1, which secretes the effectors EsxA and EsxB and promotes disruption of the phagosomal membrane, allowing bacteria to access the host cytosol [250]. The NAP EspR binds the *espACD* promoter (Rv3616c-Rv3614c) and activates expression of ESX-1 components required for EsxA/EsxB secretion [251,252]. EspR forms dimers through its C-terminal domain, and structural and biophysical analyses indicate that these dimers assemble into tetramers capable of bridging DNA fragments. This architectural activity promotes long-range DNA contacts at loci such as *espACD* [253]. EspR depletion markedly reduces *espACD* expression and ESX-1 activity, demonstrating that this NAP directly controls the escape machinery [252]. Remarkably, EspR is itself an ESX-1 substrate, providing a feedback connection between nucleoid regulation and the secretion system it helps activate [252].

A different mechanism is illustrated by the gain-of-function HUα E38K/V42L mutant in the commensal K-12 *E. coli* strain. This mutation increases chromosome compaction and confers an invasive phenotype, with bacteria reaching the cytoplasm rather than remaining within the phagosome [14]. The phenotype results from derepression of the *hlyE* promoter, which encodes the pore-forming toxin hemolysin E. The HU mutation induces positive supercoiling at this promoter, counteracting its repression by H-NS and promoting *hlyE* expression [177,254].

## 5. Role of NAPs in Persistence, Tolerance and Antibiotic Resistance

### 5.1. Persistence and Resistance

Antibiotic exposure represents a major stress that pathogens must withstand during infection, whether they are administered therapeutically or produced by competing microorganisms. Bacteria can survive this stress through distinct but overlapping mechanisms, including persistence, tolerance and resistance. Persistence arises from phenotypic heterogeneity within genetically identical populations, where a subpopulation of dormant, non-growing cells survives antibiotic exposure, immune pressure and metabolic stress without carrying any resistance mutations [255]. Tolerance refers more broadly to the ability of bacteria to survive antibiotic exposure without an increase in the minimum inhibitory concentration (MIC), whereas resistance results from horizontally acquired genes or *de novo* mutations that reduce antibiotic susceptibility by altering drug targets or limiting antibiotic accumulation within the cell [256]. NAPs have been implicated in these processes through their roles in dormancy, stress adaptation and regulation of antibiotic susceptibility.

In *M. tuberculosis*, the NAP NapM links nucleoid regulation to persistence by controlling chromosome replication. Under unfavorable conditions, NapM binds DnaA and inhibits replication initiation, thereby promoting a slow-growing, replication-arrested state under stress [257]. Lsr2 provides a complementary link between oxygen availability and persistence. Within granulomas, progressive oxygen limitation drives the bacterium into the non-replicating, drug-tolerant state that underlies latent infection, so that the ability to enter and exit anaerobic conditions supports long-term survival in the host [258]. In *M. tuberculosis*, Lsr2 acts as a global transcriptional regulator of genes involved in adaptation to changing oxygen levels. Its deletion causes a severe defect in anaerobic adaptation and a significant reduction in viability during oxygen depletion, while also delaying resuscitation once oxygen is restored. Consistent with these defects, the Δ*lsr2* mutant establishes an early infection in mice but is severely impaired in persistent lung infection [148]. Loss of *lsr2* likewise impairs survival under hypoxia in *Mycobacterium smegmatis* [108].

Persistence can also be shaped by post-translational regulation of NAPs (Table 2). In *M. smegmatis*, isoniazid exposure selects large- and small-colony variants, and HupB is required for the formation of both, whereas the K86R substitution specifically abolishes the formation of the small-colony variant, indicating that modification of this residue contributes to the establishment of a drug-tolerant state [259]. K86 acetylation alters HU–DNA binding, shifting it from DNA bending and looping toward the formation of extended, rigid filaments, potentially linking HU modification to nucleoid changes associated with persistence [260]. In *B. subtilis*, the HU homologue HBsu is also acetylated on seven lysines, with site-specific effects on nucleoid compaction [261]. These architectural differences are associated with distinct survival phenotypes: acetylation-mimic mutants are killed faster by kanamycin and vancomycin but form more persisters during prolonged exposure, whereas deacetylation mimics take several hours to resume growth after antibiotic removal. The importance of nucleoid compaction for persistence is supported by the K37R mutant, which does not alter compaction and recovers from antibiotic exposure like the wild type. These findings suggest that HU-dependent changes in nucleoid architecture contribute to both entry into and exit from the persistent state [261].

NAPs can also directly influence antibiotic susceptibility and resistance by regulating resistance determinants and cellular responses to antibiotic-induced damage. In *A. baumannii*, colistin treatment failure has been directly linked to inactivation of H-NS, with an ISAba125 insertion within the *hns* locus converting a susceptible clinical isolate into a highly resistant variant [262]. Restoring *hns* reversed resistance, demonstrating that H-NS normally constrains transcriptional pathways that remodel lipid A, the LPS component targeted by colistin. Loss of H-NS prominently upregulates *eptA*, encoding a phosphoethanolamine transferase that increases lipid A modification independently of the classical *pmrCAB* pathway [262].

In *P. aeruginosa*, Fis directly represses *prtN*, the master activator of pyocin synthesis, by binding its promoter, while mutating the Fis-binding site abolishes this repression [263]. In *fis* mutants, fluoroquinolone-induced genotoxic stress triggers uncontrolled pyocin production and self-lysis, whereas Fis-mediated repression of these bacteriocins prevents antibiotic-induced self-destruction [263]. HU provides a complementary mechanism through nucleoid remodeling. In *M. smegmatis*, antibiotic exposure induces rapid nucleoid shrinkage, a protective architectural response that preserves chromosomal integrity and facilitates recovery after removal of the antibiotic [264]. Other NAPs regulate antibiotic susceptibility through resistance determinants. In *M. abscessus*, Lsr2 positively regulates genes encoding enzymes that modify aminoglycosides and macrolides, while deletion of *lsr2* correlates with increased susceptibility to these antibiotics during macrophage infection [109]. In *M. smegmatis*, Lsr2 conversely represses the *iniBAC* operon, whose constitutive expression in *lsr2* mutants confers increased tolerance to ethambutol [265].

Finally, resistance can also result from the export of antibiotics before they reach their intracellular targets. In *M. tuberculosis*, HU enables bacteria to resist extreme concentrations of rifampicin and isoniazid. *hu* knockouts are rapidly killed by drug levels far below the MIC, whereas wild-type and complemented strains survive prolonged exposure to concentrations up to 400x MIC. This tolerance depends on transcriptional control of efflux systems, which are downregulated in *hu* mutants and upregulated upon HU overexpression, increasing INH MICs by 3-fold [226].

### 5.2. Virulence In Vivo and Therapeutic Targeting of NAPs

The contribution of NAPs to virulence becomes more evident in animal infection models, where these effects translate into clear phenotypic consequences. In *M. abscessus*, Lsr2 is indispensable for full virulence in zebrafish and mice [228]. Similarly, HU is required to maintain infection in mice by *M. tuberculosis* [266]. The contribution of HU is even more striking in *F. tularensis*, where deletion of *hupB* abolishes lethality in mice even at 10^6^ CFU, and the surviving animals resist a subsequent challenge with the wild-type strain [234]. Point substitutions within the HU-DNA interface have the same effect, attenuating bacteria while protecting all challenged mice [267]. These observations provide a rationale for targeting NAPs therapeutically, with several approaches now emerging to interfere with their DNA interactions or higher-order functions.

In *M. tuberculosis*, solving the crystal structure of the N-terminal domain of MtHU identified a targetable region within the HU-DNA interface. Stilbene derivatives binding this region inhibit HU association with DNA, disrupt nucleoid architecture and reduce bacterial growth, while inducing transcriptional changes that resemble HU depletion [268]. Consistent with this mechanism, HU depletion increases the sensitivity of *M. tuberculosis* to isoniazid, while the stilbene-derived inhibitor SD1 potentiates isoniazid activity, including in THP-1 macrophages [226]. In another approach, structure-guided docking identified drug-like scaffolds predicted to bind a partially hydrophobic pocket surrounding the MtbHU DNA-binding domain, suggesting additional sites for HU inhibitor development [269]. In *H. pylori*, the epigallocatechin gallate (EGCG), a major green tea polyphenol, directly binds the DNA-binding saddle of Hup, disrupting dimerization and DNA interaction, thereby altering nucleoid organization and stress tolerance that sustains chronic gastric infection [270]. An alternative strategy exploits HU-protein interactions. The bacteriophage SPO1 protein Gp46 binds the DNA-binding surface of HU and displaces DNA, disrupting HU function. In *B. subtilis*, Gp46 expression causes nucleoid segregation defects and filamentation, while in *F. tularensis*, it abolishes HU DNA binding, reduces bacterial growth and strongly impairs intracellular proliferation in macrophages, recapitulating features of HU depletion [271,272]. The ability of Gp46 to recognize conserved determinants in HU proteins from diverse pathogens suggests that HU-protein interactions could provide a broader structural framework for targeting this NAP.

DNABII proteins, including HU and IHF, can also be targeted through their extracellular role in biofilm architecture. Monoclonal antibodies directed against the DNA-binding surface of DNABII proteins recognize epitopes exposed when these proteins are unbound to eDNA. By trapping DNABII proteins in this “off” state, the antibodies progressively extract additional DNABII molecules from the eDNA matrix, triggering rapid biofilm collapse [143]. This strategy allows disruption of biofilms formed by several pathogens, including *Staphylococcus aureus*, *P. aeruginosa*, *Burkholderia cenocepacia* and *Moraxella catarrhalis.* In *P. aeruginosa*, antibody treatment showed significant activity against lung biofilms and nearly complete clearance when combined with tobramycin, whereas this antibiotic alone had little effect on biofilm-embedded bacteria [143]. This concept was further supported in cystic fibrosis sputum, where IHF was detected in most samples and anti-IHF antibodies promoted sputum dissolution, with an even stronger effect when combined with DNase [273].

Beyond HU and IHF, other NAPs have also emerged as potential therapeutic targets. Lsr2 was identified as a target of the repurposed anti-asthmatic drug Zafirlukast using a fluorescence polarization assay monitoring Lsr2 binding to AT-rich DNA [274]. Zafirlukast directly binds Lsr2 and inhibits its association with DNA, resulting in derepression of Lsr2-regulated genes and dose-dependent inhibition of mycobacterial growth. A recent study showed that Zafirlukast also has bactericidal activity against replicating *M. abscessus*. Notably, treatment induced rapid DNA condensation within one hour, followed by progressive alterations in bacterial morphology and loss of viability. Although not directly attributed to Lsr2 inhibition, the DNA phenotype is consistent with Lsr2 contributing to the observed nucleoid reorganization [275].

Despite their conceptual appeal, targeting NAPs faces several important challenges. The intracellular delivery of small molecules to the bacterial nucleoid may be limited by restricted permeability and active efflux systems, while the conservation of NAPs across bacterial species raises concerns about target specificity and potential negative effects on commensal bacteria. Their regulatory networks may also be sufficiently plastic to allow compensation through changes in DNA topology, altered expression of other NAPs, or other regulatory mechanisms. Moreover, because NAPs control broad transcriptional programs, their inhibition could have pleiotropic effects that can be difficult to predict and may affect bacterial physiology beyond the intended therapeutic target. Finally, most approaches described above remain at the proof-of-concept stage, with limited validation in infection models and no clinically approved NAP-targeting inhibitors to date.

## 6. Conclusions and Perspectives

By following pathogens from their environmental reservoirs to intracellular compartments of the host, this review highlights the central role for NAPs in linking nucleoid organization to bacterial adaptation and virulence. The bacterial chromosome is not a passive repository of genetic information but also a dynamic structure whose organization responds to environmental and host-derived cues. One of the most striking examples comes probably from *E. coli* K-12, where a subtle change in the sequence of the global chromosome organizer HU is sufficient to convert a commensal strain into an invasive one, without acquisition of new genetic material [177]. Thus, pathogenicity depends not only on the genetic content, but also on how its chromosome is organized and regulated.

NAPs can influence this process even before host contact, through the silencing and controlled expression of horizontally acquired genes, linking genome evolution to pathogenic potential. Once pathogens encounter host environments, NAPs can respond directly to changes in temperature, pH, osmolarity, nutrient availability, and oxygen through changes in their DNA-binding properties, coupling physicochemical signals to chromosome organization and gene expression. The chromosome therefore acts as an interface between environmental conditions and bacterial physiology. Throughout infection, NAPs further coordinate the expression of virulence programs, from secretion systems and adhesins to biofilm formation and intracellular persistence. Their ability to combine structural, regulatory, and environmental sensing functions allows changes in nucleoid organization to be directly connected to pathogenicity.

Several limitations should nevertheless be considered. NAPs are highly pleiotropic, making it difficult to assign the phenotype of a NAP mutant to a single regulatory pathway, as illustrated by the *hns* mutant of *V. cholerae*, whose colonization defect cannot be separated from its general fitness defect, or by Lsr2, whose protective effects against DNA damage differ between *M. abscessus* and *M. tuberculosis*. Moreover, mechanistic evidence remains limited across bacterial species, with most detailed studies focusing on enterobacteria. Important questions therefore remain about how NAPs interact, compete, or cooperate on the chromosome, and how post-translational modifications tune their activities [68,69], and how nucleoid architecture changes across ecological niches and stages of infection. Addressing these gaps will require future studies combining structural biology, single-cell imaging, advanced transcriptomics and chromosome conformation capture (Hi-C and Micro-C) across a wider range of pathogens.

These properties also make NAPs potential therapeutic targets. Inhibition of a single NAP could affect several virulence programs simultaneously, as illustrated by stilbene derivatives that disrupt the HU-DNA interface [268]. However, their pleiotropic functions may also complicate therapeutic targeting, while challenges related to intracellular delivery, target specificity, and compensatory mechanisms remain to be addressed. An alternative approach may be to exploit NAP-dependent attenuation: in *F. tularensis*, loss of *hu* not only reduces virulence but also protects mice against subsequent challenge with a virulent strain [234]. Such findings raise the possibility of exploiting NAP-dependent attenuation not only for antimicrobial development but also as a strategy for generating attenuated vaccine strains.

## Figures and Tables

**Figure 1:**
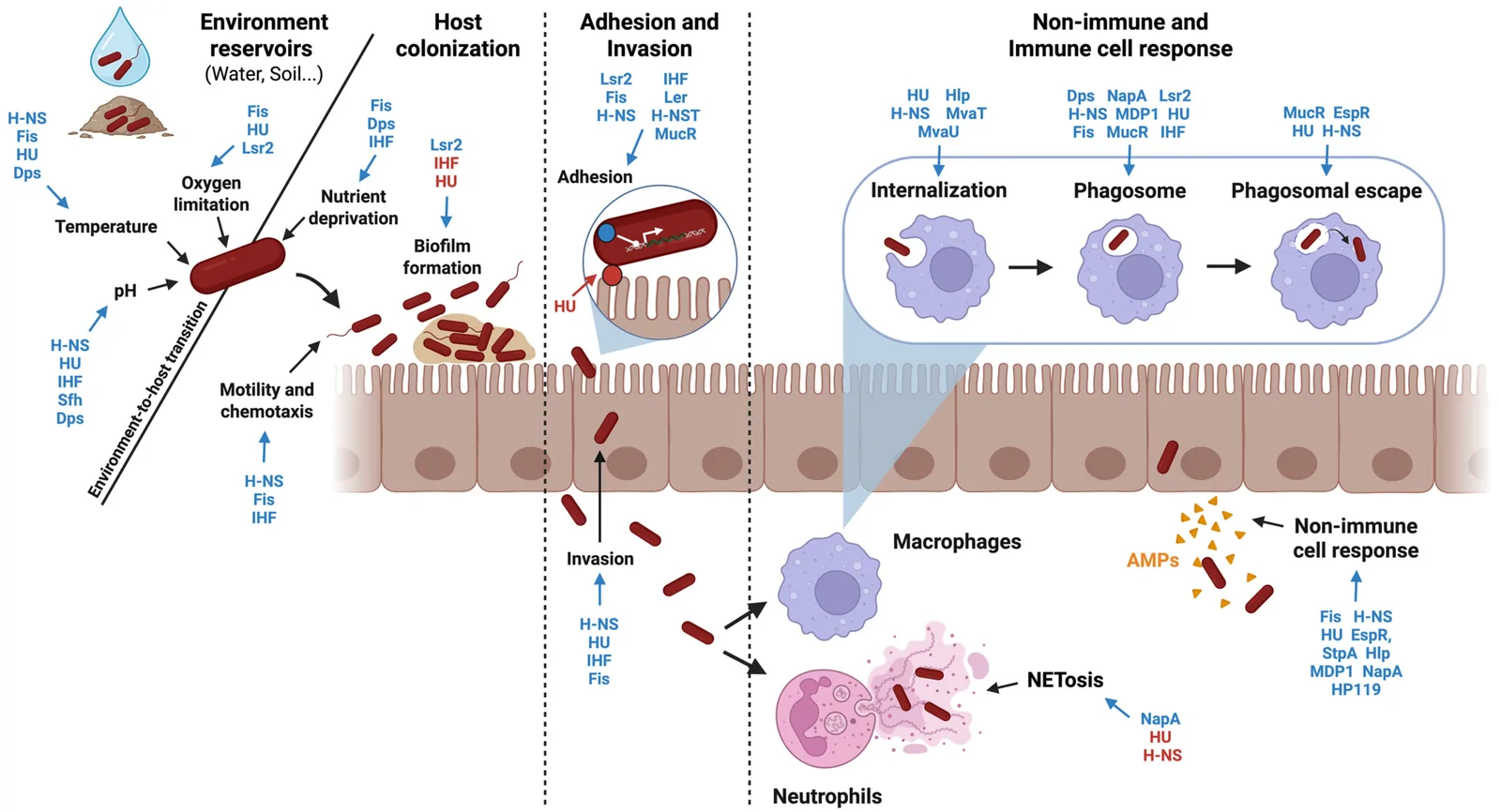
Role of Nucleoid-associated proteins throughout the infectious cycle. NAPs contributing to each stage of infection are indicated in blue, except for secreted NAPs in red. In environmental reservoirs and during host transition, pathogens face fluctuations in temperature, pH, oxygen and nutrient availability, which NAPs integrate into modifications of chromosome organization and gene expression (Section 2.2). Colonization involves biofilm formation, motility and chemotaxis (Section 3.2 and Section 3.3), followed by adhesion and invasion of epithelial cells (Section 3.4 and Section 3.5). Bacteria consequently encounter epithelial cells producing antimicrobial peptides (AMPs, orange), neutrophil-mediated extracellular traps (NETosis), and macrophages, with NAPs regulating bacterial internalization, phagosomal survival and escape (Section 4). NAPs may act positively or negatively depending on the species and cellular context. (Created in https://BioRender.com).

**Table 1:** Overview of nucleoid-associated proteins across pathogens.

Mechanism of Action	Representative NAP	Other Names	Bacterial Genus or Species Covered in This Review
Bending	HU	Huα/Huβ	*Enterobacteria*, *Helicobacter pylori*, *Vibrio cholerae*, *Acinetobacter baumannii*, *Porphyromonas gingivalis*
		HUBs	*Bacillus subtilis*
		HupB	*Mycobacteria; Francisella tularensis*
	IHF	IhfA/IhfB	*E. coli*, *Salmonella*, *K. pneumoniae*, *Shigella*, *S. aureus*, *P. aeruginosa*
	Fis		*E. coli*, *Salmonella*, *Serratia*, *EPEC*, *Shigella*, *Erwinia*, *Yersinia*, *P. aeruginosa*
Bridging	H-NS		*Enterobacteria*, *H. pylori*, *Vibrio spp.*, *Salmonella*, *K. pneumoniae*, *Shigella*, *A. baumannii*
		StpA	*E. coli*, *Salmonella typhimurium*
		Sfh	*Shigella flexneri*
		Hha/YdgT	*V. cholerae*, *Salmonella SPI-1*, *E. coli*
		Ler	EHEC
		Hfp	UPEC
		Lsr2	*Mycobacteria* spp.
		Rok	*Bacillus subtilis*
		MvaT/MvaU	*Pseudomonas* spp.
		HP119	*Helicobacter pylori*
		Pmr/TurA/TurB	*Enterobacteria* (plasmids)
		MucR	*Brucella* spp.
	EspR		*Mycobacteria* spp.
	NapM		*Mycobacterium tuberculosis*
Wrapping	Dps		*E. coli*, *Salmonella*, *Brucella abortus*
		NapA	*Helicobacter pylori*
		MrgA	*Bacillus subtilis*
	HU	Hlp	*Mycobacterium smegmatis*, *M. bovis*
		MDP1	*Mycobacteria* spp.

**Table 2:** Summary of post-translational modifications of NAPs regulating infection.

NAP	Type of PTM	Environmental/Physiological Trigger	Functional and Biological Consequences
H-NS	Phosphorylation (Ser42)	Temperature-dependent signaling	Maintains silencing of cold-inducible prophage genes in *S. oneidensis*.
H-NS	Multi-site phosphorylation (S45, Y61, S78, S84, T86, T106)	Intracellular stress during infection	Promotes H-NS dissociation from DNA via a dimer-dependent mechanism; activates T3SS genes in *S. Typhimurium*.
H-NS	Acetylation	Metabolic stress (amino acid)	Reduced DNA binding; readily displaced by counter-silencers, activating T3SS/T6SS loci in *Edwardsiella*
HupB	Acetylation (K86)	Antibiotic exposure (isoniazid), Persistence-associated stress	Shifts HU–DNA binding from bending/looping to rigid filament formation; supports persistence-related nucleoid remodeling in *M. smegmatis*.
HBsu	Acetylation (multiple lysines)	Antibiotic exposure (Kanamycin)	Modulates DNA compaction and antibiotic survival dynamics in *B. subtilis*.

**Table 3:** Overview of secretion systems regulation by NAPs and their involvement in the infection process.

Secretion Systems		NAP Regulators		Functions				Pathogens
Type	Activators	Negative Regulators	Positive Regulators	Colonization	Adhesion	Invasion	Intracellular Survival	
III	EsxA	MvaT MvaU				x		*P. aeruginosa*
	SPI-2	H-NS Hha YdgT	IHF Fis				x	*Salmonella*
	Ler	H-NS Hha	IHF Fis		x	x		*E. coli* (EPEC)
	ExsA VtrA	H-NS				x		*V. parahaemolyticus*
	SPI1	H-NS IHF	HU			x		*Salmonella* *enterica*
	VirF VirB	HNS	IHF Fis			x		*Shigella*
IV	VirB BabR	MucR					x	*Brucella*
VI	T6SS2	H-NS		x	x			*V.* *parahaemolyticus*
	hcp	H-NS		x	x			*K. pneumoniae*
	VflT6SS2		IHF	x				*Vibrio fluvialis*
	T6SS1 T6SS2		HU	x				*Enterobacter* *cloacae*
	SPI-6	H-NS		x				*S. enterica*
VII	ESX 1		EspR				x	*M. tuberculosis*

## Data Availability

No new data were created or analyzed in this study. Data sharing is not applicable to this article.
